# Supplementary figures and images for: Downregulated miRNA-491-3p accelerates colorectal cancer growth by increasing uMtCK expression
Source: PeerJ. 2022 Nov 28;10:e14285. doi: 10.7717/peerj.14285 (PMC9744150; doi:10.7717/peerj.14285)

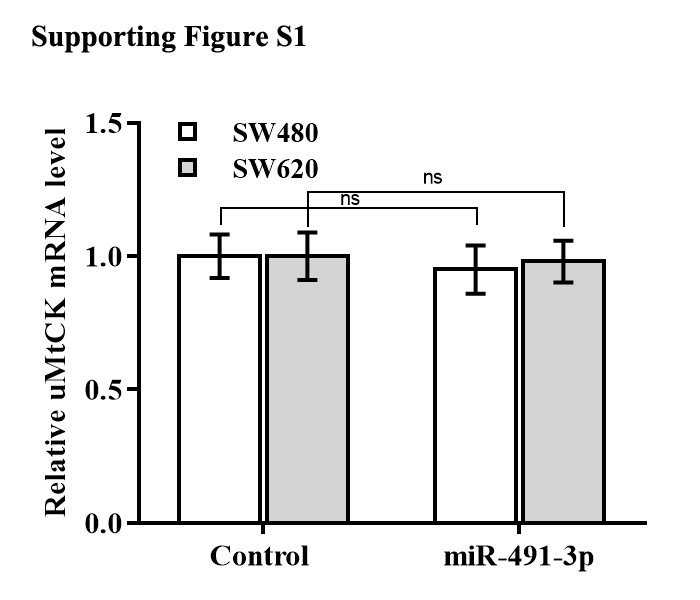

Supplement: Supplemental Information 1 [file peerj-10-14285-s001.jpg]

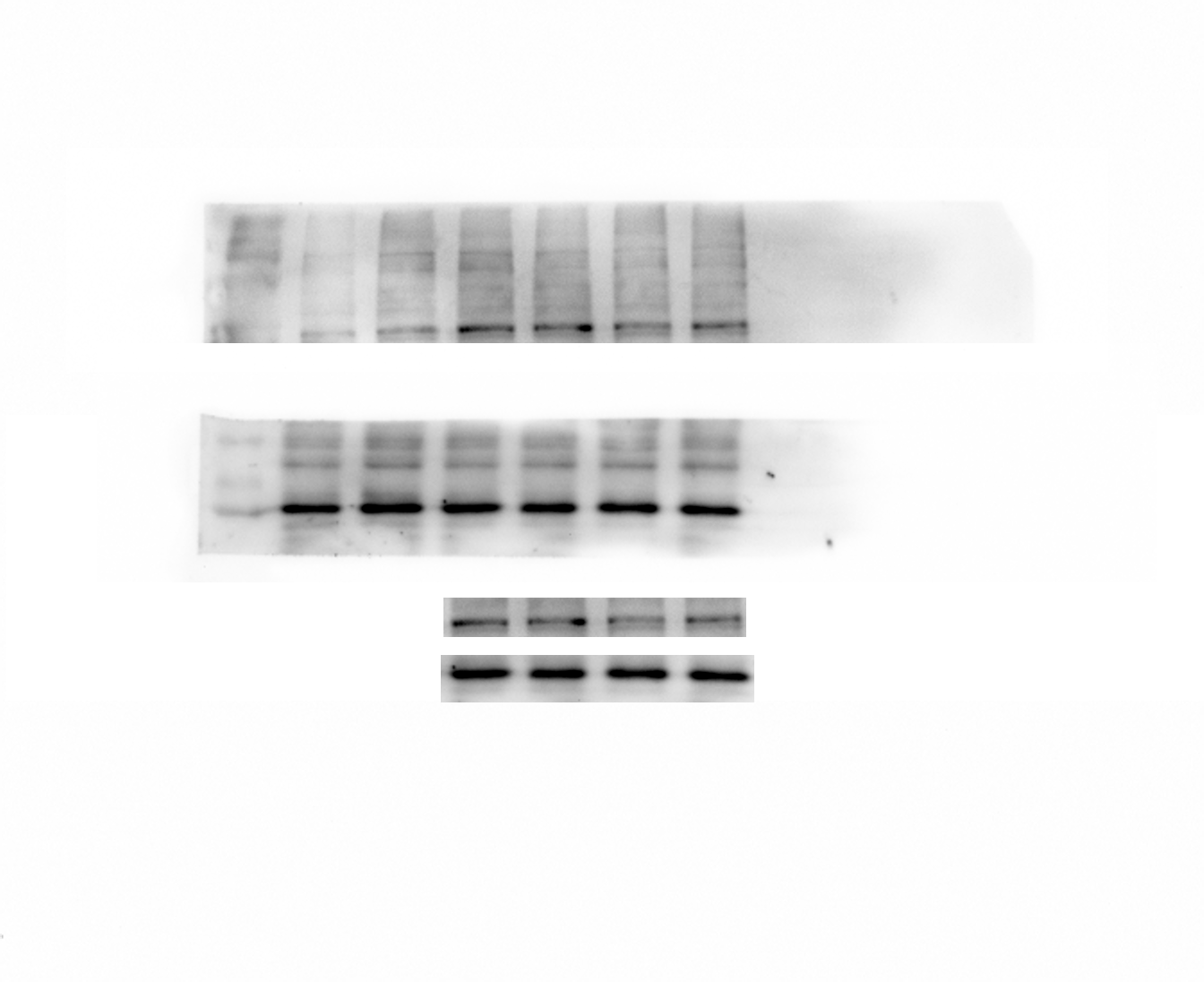

Supplement: Supplemental Information 7 [file peerj-10-14285-s007.tif]
